# Behavioral and pharmacological characterization of planarian nociception

**DOI:** 10.3389/fnmol.2024.1368009

**Published:** 2024-05-01

**Authors:** Guillaume Reho, Yannick Menger, Yannick Goumon, Vincent Lelièvre, Hervé Cadiou

**Affiliations:** CNRS UPR 3212, Institut des Neurosciences Cellulaires et Intégratives, Centre National de la Recherche Scientifique and Université de Strasbourg, Strasbourg, France

**Keywords:** planarians, nociception, TRPA1, morphine, NSAID, RNA interference, meloxicam

## Abstract

**Introduction:**

Pain mostly arises because specialized cells called nociceptors detect harmful or potentially harmful stimuli. In lower animals with less convoluted nervous system, these responses are believed to be purely nociceptive. Amongst invertebrate animal models, planarians are becoming popular in a wide range of pharmacological and behavioral studies beyond the field of regeneration. Recent publications led the way on pain studies by focusing on nociceptive behaviors such as the ‘scrunching’ gait displayed under various noxious stimuli, as opposed to the ‘gliding’ gait planarians usually adopt in normal conditions.

**Methods:**

In this study, we adapted commonly used nociceptive tests to further explore nociception in planarians of the species *Girardia dorotocephala*. By using behavioral analysis in open fields and place preferences, we managed to set up chemical, thermal and mechanical nociceptive tests. We also adapted RNA interference protocols and explored the effects of knocking down TRPA1 ion channels, one of the main effectors of chemically and thermally-induced nociceptive responses in vertebrates.

**Results:**

Consequently, we demonstrated the reliability of the scrunching gait in this planarian species, which they displayed in a dose-dependent manner when exposed to the irritant AITC. We also showed that suppressing the expression of TRPA1 ion channels completely suppressed the scrunching gait, demonstrating the involvement of TRPA1 nociceptors in this nociceptive reaction. Besides, we also explored the effects of two common analgesics that both displayed strong antinociceptive properties. First, morphine reduced the chemically-induced nociceptive scrunching gaits by more than 20% and shifted the $EC_{50}$ of the dose–response curve by approximately 10 μM. Secondly, the NSAID meloxicam drastically reduced chemically-induced scrunching by up to 60% and reduced heat avoidance in place preference tests.

**Discussion:**

Thus, we managed to characterize both behavioral and pharmacological aspects of *G. dorotocephala*’s nociception, further developing the use of planarians as a replacement model in pain studies and more globally the study of invertebrate nociception.

## 1 Introduction

Planarians are free-living freshwater flatworms that are known for their remarkable regenerative capabilities, which made them highly popular amongst the regeneration and development fields of biology (Elliott and Alvarado, 2018). However, the planarian model has also been getting more and more popular amongst pharmacologists and toxicologists (Pagán, 2017). In the last decade, flatworms proved to be a very practical model for both environmental toxicology and high-throughput screenings: they reproduce quickly, need a low-cost maintenance, and are soft-bodied, allowing for most molecules to passively diffuse through the animal without the need of invasive methods (Wu and Li, 2018; Ireland et al., 2020). Additionally, they proved not only to be helpful for toxicology (e.g., observing death of individuals), but also useful in the field of neurotoxicology, in which planarian behavior could be more precisely observed, such as in thermotaxis or with gaits they display under various drugs or toxicant exposure (Hagstrom et al., 2015). Nociception, as defined by the IASP (Terminology | International Association for the Study of Pain, 2023), is “the neural process of encoding noxious stimuli,” for which “consequences of encoding may be autonomic (e.g., elevated blood pressure) or behavioral (motor withdrawal reflex or more complex nocifensive behavior).” Even if nociception is often presented through the prism of mammalian physiology, it is also particularly relevant to explore nociception in invertebrates. In the light of the Russell’s and Burch’s 3R principles [see Hubrecht and Carter, 2019], there is a need for reliable replacement models, especially in pain studies. While worms do not represent a non-animal replacement model, studying planarian nociception is in itself also a step towards a better understanding of invertebrates’ “pain” defense systems. Planarians constitute a promising model for nociception and could also be implemented as a first-line *in vivo* drug screening to replace rodents, which are predominantly implemented in pain studies (Mogil, 2009). Invertebrates’ nervous system also encode noxious stimuli, and the neural processes of this encoding is well documented for a small handful of animal models (Burrell, 2017). In most other invertebrate models, nociceptors descriptions or nociceptive reactions descriptions can be found, but full nociceptive system descriptions are scarce. Unfortunately, even though planarian behavior has been described for centuries, only a few scarce studies observing nociceptive behaviors can be found. Moreover, these few studies only described nociceptive behavior by how they look like, without properly characterizing and quantifying any specific gait, thus leading to various interpretations and unclear definitions (Reho et al., 2022). One particular tipping point of this research field is a study by Cochet-Escartin et al. from 2015 that meticulously characterized one of the nociceptive gaits observed in planarians called ‘scrunching’ (Cochet-Escartin et al., 2015). Scrunching is a muscular gait in which planarians loop through contractions and elongations while also switching their anchor on the substrate, allowing them to push themselves using their tail and to pull themselves using their head. While the frequency, the locomotion velocity or the scrunching-inducer can differ, it seems to be an ubiquitous gait in planarians. Scrunching is opposed to the normal smooth ‘gliding’ gait of planarians, which is a continuous beating of ciliated cells present on the ventral side of the animals, and helped by continuous production of mucus from rhabdite cells (Rompolas et al., 2013). Besides scrunching, other nociceptive gaits have been exploited in former studies – such as ‘C-shapes’ or ‘Corkscrew’ – but none are sufficiently well characterized to be consensually used (Reho et al., 2022). It has to be noted that these other gaits often appear altogether in a relatively chaotic fashion, with no clear distinctions between one or the other, and are often described all together as ‘seizure-like activity’ (Reho et al., 2022). On the other hand, these definitions issues did not hinder new research on nociceptive planarian behavior to emerge with alternative types of tests, such as thermotaxis or thigmotaxis (Inoue et al., 2015). These tests do not involve specific gaits but utilize place preference, and mostly avoidance of noxious stimuli (e.g., hot water or sharp surfaces). Altogether, these recent studies were also able to link causal relationships between nociceptive reactions and the expression of the common nociceptor transducers TRP receptors (Arenas et al., 2017; Sabry et al., 2019). The knockdown of these receptors in *Dugesia japonica* and *Schmidtea mediterranea* greatly reduced heat avoidance and nociceptive gaits induced by irritant chemicals. This evidence led us to believe that planarians could constitute an interesting model to study the basis of nociception and to screen for antinociceptive molecules. With this in mind, we developed a full battery of behavioral tests in order to wholly characterize stimuli-dependent nociception in the planarian species *Girardia dorotocephala* (Gd). Gd is one the historically most studied planarian species. Their bigger size compared to other commonly studied planarian species allowed us efficient behavioral observations and their gaits showed to be highly reproducible. We also confirmed the involvement of TRPA1 ion channels in chemical nociception. Besides, it is for the first time, to the best of our knowledge, that the modulation of these nociceptive behaviors by common analgesic drugs - such as opioids or NSAIDs - are described.

## 2 Materials and methods

### 2.1 Animals

*Girardia dorotocephala* (Gd) were bought from Carolina Biological (NC, United States) in 2015 and have been maintained in our laboratory ever since. They were kept in glass containers of approx. 4 L of Volvic mineral water (Volvic, France) at constant 21°C and exposed to a 12 h:12 h light–dark cycle. They were fed beef liver twice a week. The containers were then cleaned, and the water changed. Animals were starved for 5 to 10 days prior to experimental testing. Animals were tested at least 2 h after the beginning of the dark phase. Outside of the recording chamber, they were manipulated under low intensity red light at wavelengths known to induce no inherent behavioral response (Paskin et al., 2014).

### 2.2 Chemicals

To induce nociceptive scrunching behavior, we used various concentrations of Allyl isothiocyanate (AITC, CAS:57–06-7, Sigma-Aldrich), ranging from 5 to 200 μM. AITC is an irritant, responsible for the pungency of mustard, horseradish and wasabi, and a potent TRPA1 receptor agonist (Bandell et al., 2004). It was already used in former studies for its high efficiency to induce scrunching in planarians (Arenas et al., 2017; Sabry et al., 2019). AITC oil was mixed in DMSO (CAS:37–38-5, Sigma-Aldrich) to allow final solubilization in freshwater. In final concentrations, DMSO never reached more than 0.1%. At these concentrations, DMSO does not induce any toxicity or scrunching on its own (Pagán et al., 2006; Sabry et al., 2019). The first analgesic we used was morphine HCl (CAS:57–27-2, Francopia) at either 1, 10 or 20 μM, diluted in Volvic mineral water (Volvic, France). The second analgesic we used was meloxicam (sodium salt hydrate form, CAS:71125–39-8, Sigma-Aldrich), a common non-steroidal anti-inflammatory drug (NSAID) at either 1, 10 or 100 μM, also diluted in Volvic water.

### 2.3 Chemical tests

Chemical tests To observe the behavior of planarians in an open field, we used a 14.5 cm diameter glass petri dish filled with 50 mL of solution (Volvic water ± chemical of interest). An individual worm was placed in the releasing device (inverted tip) for 2 min after the chamber’s door was closed (see below). A gentle hydraulic push was then used to expel the worm from the tip into the arena and a five-minutes video recording immediately started. Using the video recordings, behavior analysis was then performed. We manually assessed, throughout the whole recording, when the worm was moving in a ‘gliding’ gait or a ‘scrunching’ gait. If the animal did anything else than what we assessed as gliding or scrunching, it was labelled as “others.” To assess the scrunching behavior, we interpreted the movements of the worms following the descriptions of this gait by Sabry et al. that they extensively described in 2015 (Cochet-Escartin et al., 2015; Sabry et al., 2019). Further statistical analysis and graphical representations were done using the 4 last minutes of recording, leaving out the first one, because they display high amounts of undefined reactions in all conditions (see Supplementary Figure S1). We also removed from the analysis animals that displayed more than 50% of ‘other’ (undefined) reactions throughout the recording as they appeared to be stuck at the surface of the water instead of gliding to the substrate.

### 2.4 Thermal tests

**Figure 1:**
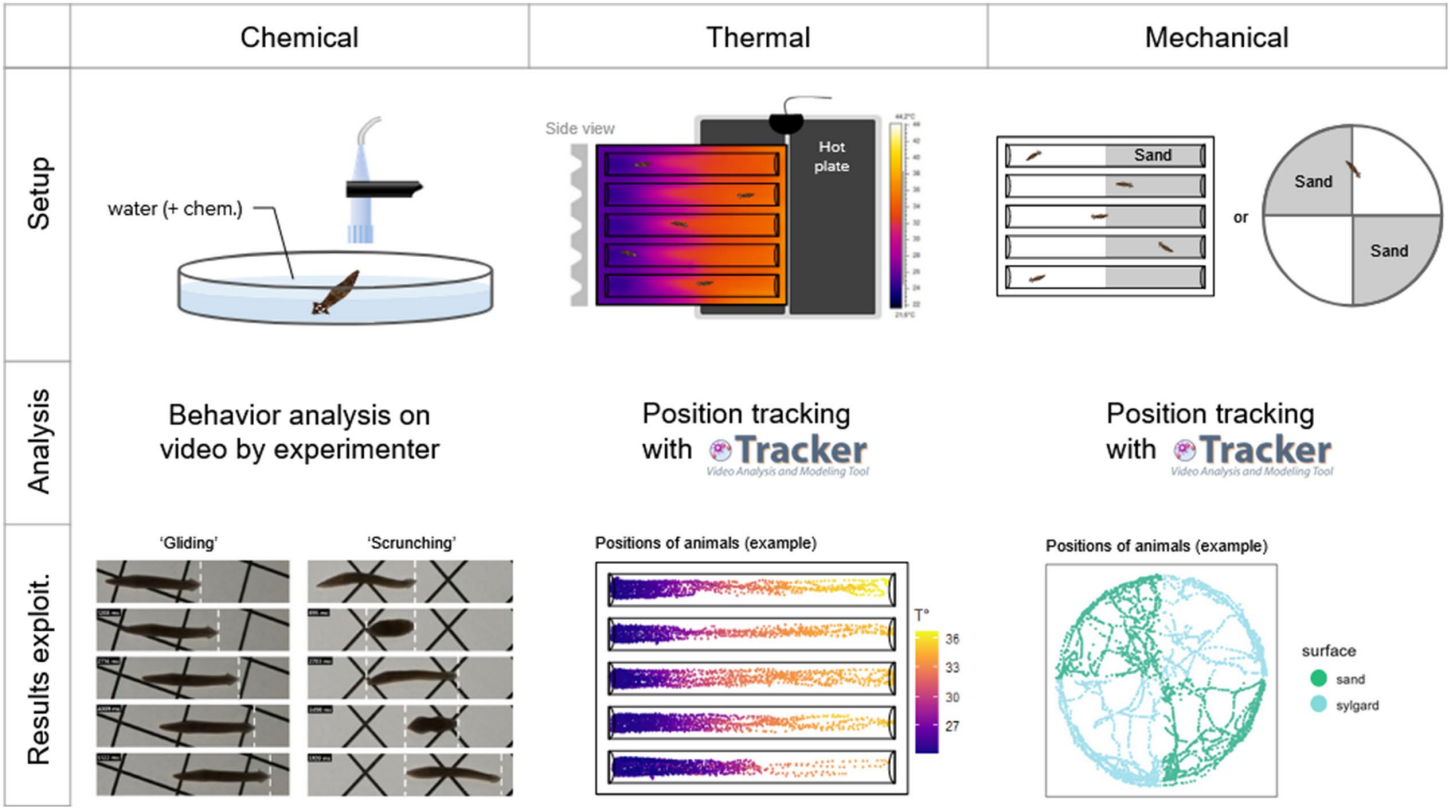
Methodology summary for each of the three types of nociception tested. For **chemical nociception**, worms were placed directly in the center of the arena with 50 mL of water, together with a chemical of interest (or nothing else for controls). They were filmed for 5 min, and their behavior was analyzed by the experimenter on video, identifying if they show a ‘gliding’ locomotion, a ‘scrunching’ locomotion, or something else. For **thermal nociception**, worms were placed in plastic ‘racetracks’ above a heating plate, resulting in a thermal gradient. They were filmed for 10 min, and their positions were tracked on video using the software Tracker (Physlet). For **mechanical nociception**, worms were placed either in an arena or a racetrack which contained sand (included in a thin layer of sylgard) in half of their surface. They were filmed for 10 min, and their positions were tracked on video using the software Tracker (Physlet).

To observe place preference in a temperature gradient, we used a custom plastic slab (16 cm long, 14.7 cm wide, 1.5 cm high) in which five linear ‘racetracks’ were dug. They were adapted from a study on planarian electrotaxis by Sabry et al. (2022). Individual racetracks were 14 cm long, 1.7 cm wide, 1 cm high and dug at a 60° angle with a round edge at the bottom. Racetracks were filled with 5 mL of solution. A flat heating mat (Lerway, 14 W), reaching 50–55°C, was placed under one half of the racetracks (see Figure 1). In approximately 20 min, the water reached the desired temperature range of 22°C to 34°C from the room temperature (RT) side to the hot side. Worms were then placed by hand in the middle of each racetrack and the video recording was set for 10 min. The temperature gradients along the tracks were recorded using a thermal camera (see below) and the values were associated with the positions of the animals. In order to delimit the hot side from the RT side in statistical analysis and graphical representations, we did not choose a set temperature as a threshold (e.g., 30°C), nor did we choose a position as a threshold (e.g., the center of the track), because they slightly differed between experiments. Thus, we chose to rescale the temperature ranges using the min-max normalization and set the threshold to 0.5. Even though this normalization meant that one virtual side of the racetrack would be bigger than the other one, we did not perform any additional correction for increased probability of a worm spending time on larger sides (see Supplementary Figure S3).

### 2.5 Mechanical tests

Another place preference test was set up using sharp surfaces to explore potential mechanical nociception. We used arenas, the same glass petri dish as in chemical tests, and racetracks, as in thermal tests. This setup was adapted from studies by Inoue et al. (2015) and Mohammed Jawad et al. (2018). In arenas, a thin layer of silicone resin (RTV 141 A, Rhodorsil Silicones) was cast and either fine sand (1–1.5 mm grains) or coarse sand (1.5-3 mm grains) was stuck into two opposite quarters of the arena (including the vertical sides of the petri dish). In racetracks, one half was covered in sylgard to stick either fine or coarse sand as well. Worms were set in the middle of each setup (on the edge of both surfaces) and were recorded for 10 min.

### 2.6 Acute and baths exposure

For both chemical and thermal tests, two types of analgesic exposures were performed. They were either applied acutely (as previously described above), or prior to behavioral experiments. For the latter, animals were placed individually in approximately 10 mL of an analgesic solution for 2 h. The experimental tests were carried out immediately at the end of the 2 h exposure without the presence of the analgesic.

### 2.7 Video acquisitions

Video acquisitions were set inside a 108x79x88cm light-tight home-made chamber built from foam panels. The inside was illuminated by a circular (31 cm Ø) infrared (850 nm) LED strip allowing the infrared-sensitive camera (Arducam IMX477) to capture the area where worms were set. A 34 cm-tall stand was set in the center to elevate the experimental zone 46 cm below the camera, which was attached to the ceiling of the chamber. The camera was controlled by a micro-computer (Raspberry Pi 4) outside the chamber. Videos were acquired at 10 frames per second. A thermal camera (FLIR A325) was also attached to the setup to precisely record the temperature that the worms were exposed to in thermal tests. Thermal recordings were processed through the camera’s associated software (FLIR ThermaCam Research, Professional edition, version 2.9).

### 2.8 Tracking

The positions of the animals were tracked using the Tracker software (Brown et al., 2023). Positions were manually tracked at 1 frame per second.

### 2.9 Statistical analysis

All statistical analysis were done using the R ‘ggpubr’ package in Rstudio (RStudio Team, 2020; Kassambara, 2023). Unless stated otherwise, all statistical tests realized were pairwise Wilcoxon test. Statistical significance was set to *p* < 5% (*), *p* < 1% (**), *p* < 0.1% (***). Results were expressed as average ± SEM, and n representing the number of animals.

### 2.10 Dose–response curve modelling

To analyze the dose–response curve parameters, a fit was modeled from a three-parameter log-logistic Hill equation using the ‘drc’ R package (Ritz and Strebig, 2016). The model was based on the following equation:


$$\mathrm{Hill}\left(x\right)=\frac{E_{\text{max}}}{1+{\left(\frac{EC_{50}}{\left[x\right]}\right)}^{n}}$$

### 2.11 RNA interference (RNAi)

**Figure 2:**
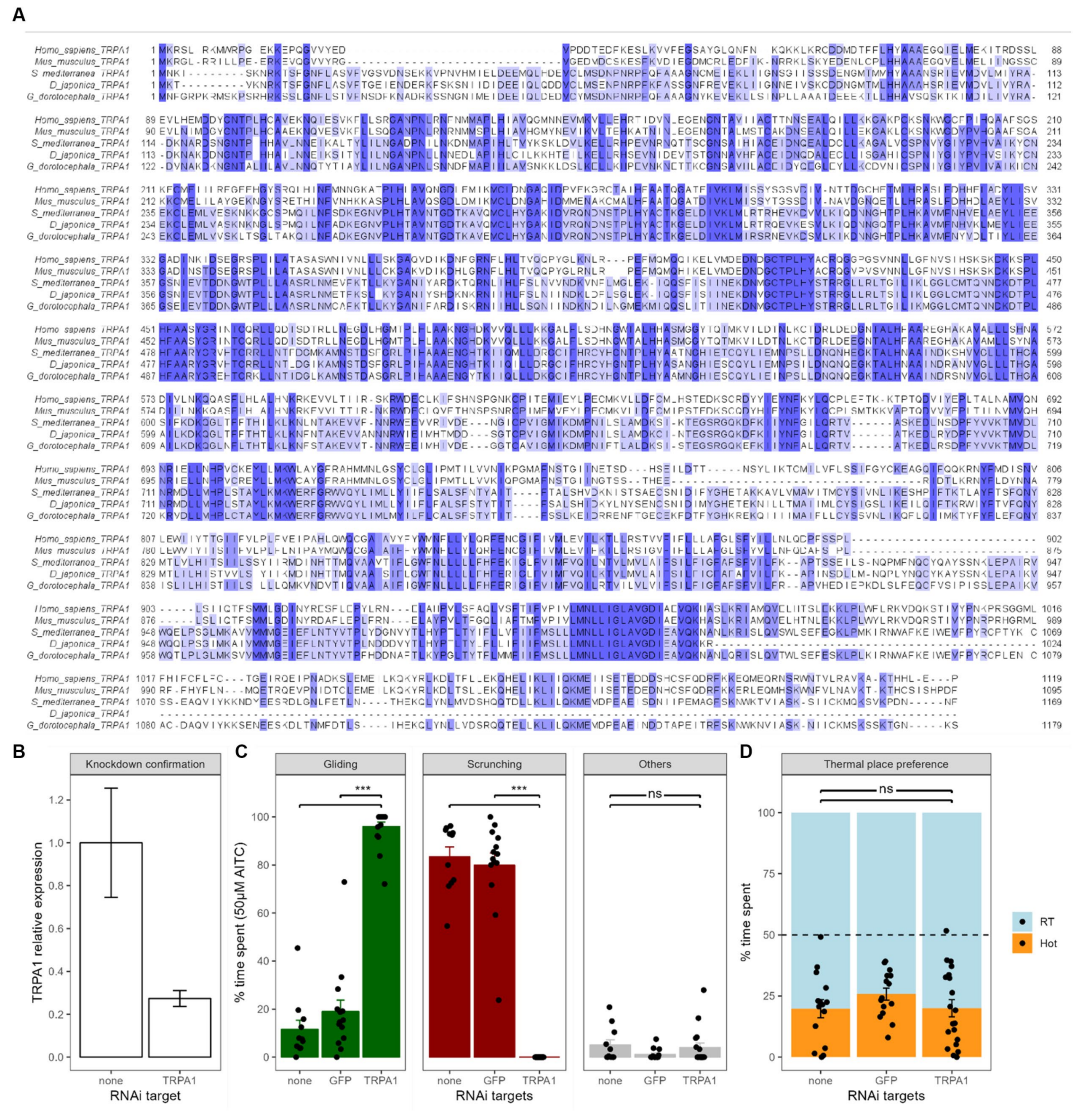
Effects of TRPA1 RNAi on chemical and thermal nociception. **(A)** The *Girardia dorotocephala* TRPA1 cDNA sequence obtained from an RNAseq blast (Rouhana, 2017) has been *in silico* transformed into the corresponding protein sequence and compared to the TRPA1 sequence of several other species, including human, mice, *Dugesia japonica* and *Schmidtea mediterranea.* Analysis was done using T-coffee scores in the Jalview software; colors show amino acid conservation. **(B)** Confirmation of knockdown by RT-qPCR. Gd-TRPA1 expression was normalized over Gd-GAPDH expression. Bars represent the mean and the error bars represent the SEM (also valid for next panels). Gd-TRPA1 expression was reduced by more than 70% (*p* < 0.01). **(C)** Effects of Gd-TRPA1 RNAi on the different behaviors of planarians in 50 μM of AITC compared to a GFP RNAi and untreated worms. Black dots represent individual animals (also valid for next panel). **(D)** Effects of Gd-TRPA1 RNAi on heat avoidance compared to a GFP RNAi and untreated worms.

The gene knockdown protocol was based on feeding planarians with *in vitro*-synthesized double-stranded RNA (dsRNA) and was adapted from the protocols by Rouhana et al., (2013) and Shibata and Agata (2018). The cDNA sequences from Gd (TR25446 for TRPA1 and TR119786 for GAPDH) were obtained from the RNA-Seq sequence published by Rouhana (2017). To confirm the Gd-TRPA1 gene identity, the corresponding cDNA sequence was blasted with NCBI’s blast tool and compared to the TRPA1 gene sequences from several other species (Figure 2A) using T-Coffee scores within the Jalview software (Waterhouse et al., 2009). There was a 30.58% identity with mice (*M. musculus*), 30.87% with humans (*H. sapiens*), 72.76% with the planarian species *S. mediterranea* and 73.49% with the planarian species *D. japonica*. Primers were designed using the Primer3 software to generate a 728 bp TRPA1 PCR fragment and a 401 bp EGFP PCR fragment (Koressaar and Remm, 2007) (Table 1). These fragments were cloned into pCRII-TOPO vectors using the TOPO TA Cloning Kit dual promoter (Invitrogen) and clones were sequenced using M13 primers (Eurofins genomics). PCR was then performed using T7 promoter-flanked primers and the plasmid templates. dsRNA was subsequently synthesized using the T7 MegaScript kit (Invitrogen). 50 μL pellets of a mix of beef liver paste containing dsRNA (0.5 μg/μL), blue food coloring dye (3%) and agarose (0.3%) was given to groups of 10–12 starved worms. They were fed pellets 3 times at 3–4 days interval. Once the pellet has been eaten, nonblue-dyed worms were systematically removed from the process. Blue-dyed worms that ingested the dsRNA were finally starved again for 7 days before experimental testing. After experimental testing, RNA samples were obtained from whole animals (*n* = 4–6 animals per time point). Tissue samples were dissociated in the guanidium isothiocyanate solution using ultra Turrax homogenizer whilst total RNA were further isolated by phenol-chloroform extraction followed by DNAseI treatment. Highly purified RNA samples were quantified using Nanodrop spectrophotometry and Reverse transcription was performed with 800 ng of total RNA (iScript cDNA synthesis kit, Biorad) and the following real-time polymerase chain reaction was set up according to the manufacturer recommendation (iQ SYBR Green Supermix, Biorad) in duplicates using a 3-step protocol (95C, 60C and 72C for 20s ea.). To quantify and validate effective knock-down of Gd-TRPA1 gene expression, two sets of primers spanning different areas of the gene of interest have been designed: one pair of primers was located outside the region corresponding to the Gd-TRPA1 dsRNA and can therefore only amplify endogenous Gd-TRPA1 mRNA; the second one targeted the region corresponding to the Gd-TRPA1 dsRNA and can therefore amplify both endogenous Gd-TRPA1 mRNA and exogenous Gd-TRPA1 dsRNA. Preliminary tests revealed that primers sets were highly specific (PCR efficiency >97%) and selective (one single peak at the expected temperature observed on melting curve). Gene expression was calculated using Gd-GAPDH house-keeping gene to normalize Gd-TRPA1 expression according to the delta–delta Ct method (2–∆∆Ct) method.

**Table 1:** Primers used in this study.

Primer	Sequence
Gd-TRPA1-Fw	TGAGTAAACCATCAAGACATAGG
Gd-TRPA1-Rev	GACCACCAACATCTCCAAGCA
Gd-EGFP-Fw	AGGACGACGGCAACTACAAG
Gd-EGFP-Rev	GTCCATGCCGAGAGTGATCC
T7-Gd-TRPA1-Fw	TAATACGACTCACTATAGGGTGAGTAAACCATCAAGACATAGG
T7-Gd-TRPA1-Rev	TAATACGACTCACTATAGGGGACCACCAACATCTCCAAGCA
T7-Gd-EGFP-Fw	TAATACGACTCACTATAGGGAGGACGACGGCAACTACAAG
T7-Gd-EGFP-Rev	TAATACGACTCACTATAGGGGTCCATGCCGAGAGTGATCC
Gd-TRPA1-qPCR-outside-Fw	TGCATATTGTCGACGAAGGGG
Gd-TRPA1-qPCR-outside-Rev	TGTCCTCGGCTACCTTCAGT
Gd-TRPA1-qPCR-inside-Fw	ACCATTTCAATTCGCCGCTG
Gd-TRPA1-qPCR-inside-Rev	TCCTCTTCATCAGTTGCTGCA
Gd-GAPDH-qPCR-Fw	TGTCTCGCTCCAATGGCAAA
Gd-GAPDH-qPCR-Rev	AGTTTCTGCGACGGACCATC

## 3 Results

### 3.1 Chemical nociception

#### 3.1.1 Chemically-induced nociception

**Figure 3:**
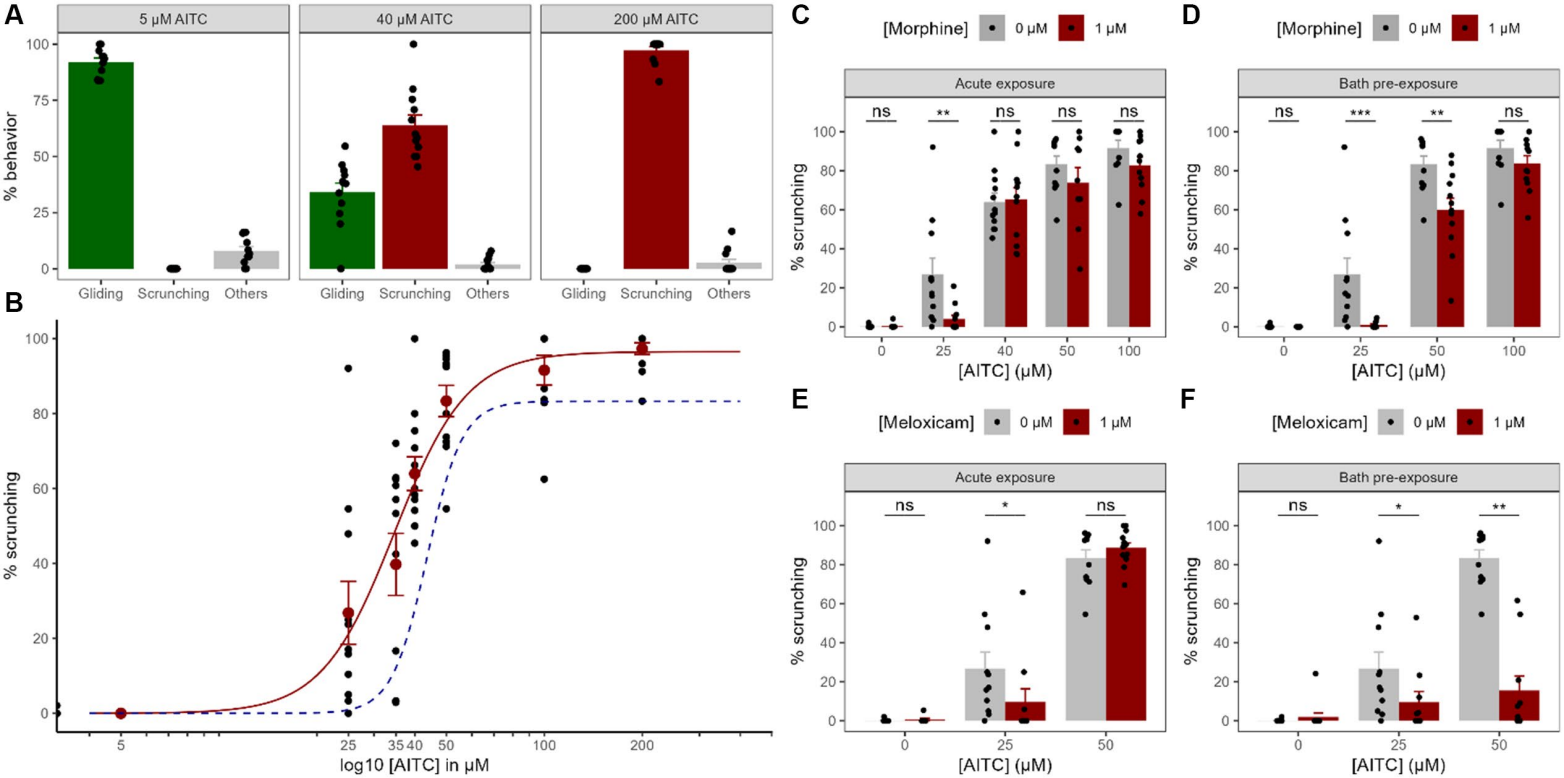
Chemical nociception and modulation by painkillers. **(A)**. Dose–response curve of the worms’ behavior exposed to AITC focused on 3 different concentrations of AITC to highlight the compensation between the percentage of time spent gliding (non-nociceptive) gait with the scrunching (nociceptive) gait. The “other” category regroups everything else that is not either gliding or scrunching, so the sum of the three groups makes 100%. **(B)**. Whole dose–response curve for the scrunching behavior. Black dots represent individual animals and red dots represent means +/− SEM. The red curve is the corresponding best fit for a Hill model. The dashed blue curve is the best fit for a Hill model corresponding to animals exposed to 1 μM of morphine for 2 h before being exposed to the same AITC concentrations visualizing the curve shift (data from panel **D**). **(C–F)** Effects of morphine **(C,D)** or meloxicam **(E,F)** on the scrunching behavior in different AITC concentrations, either acutely **(C,E)** or after a 2 h pre-exposure bath **(D,F)**. Black dots represent individual animals, bars represent the mean and the error bars represent the SEM.

In order to investigate the chemically-induced nociceptive behavior in Gd, we exposed the animals to various concentrations of AITC. When exposed to increasing concentrations of AITC, planarians behave in a very reproducible and specific muscular gait called ‘scrunching’, compensating for the gradually decreasing ‘gliding’ gait usually seen in normal conditions (see Figure 1). Without AITC, the worms showed almost no scrunching gait at all (0.1 ± 0.1%, *n* = 19, Figure 3B). Even at a very low AITC concentration of 5 μM, no scrunching gait could be seen (0 ± 0%, *n* = 11), and the worms still showed a normal gliding gait (91.9 ± 1.9%, *n* = 11, Figure 3A). At 25 μM and 35 μM, a substantially higher amount of scrunching was present, respectively 26.8 ± 8.4% (*n* = 11) and 39.8 ± 8.3% (*n* = 11). At 40 μM, the scrunching gait reached 63.9 ± 4.5% (*n* = 12) but a substantial amount of gliding gait could still be seen (34.1 ± 4.1%, *n* = 12). At 50 μM, scrunching reached a value of 83.4 ± 4.2% (*n* = 11). At 100 μM, planarians almost exclusively displayed scrunching (91.6 ± 4.0%, *n* = 10). At 200 μM, values almost reached a maximum with 97.3 ± 1.6% (*n* = 12) of scrunching, while no gliding motion could be seen anymore (0 ± 0%, *n* = 12). In order to model a dose–response curve, we used the Hill equation. The best fit was found to be modeled by $E_{\text{max}}$ = 96.52%, $EC_{50}$ = 34.65 μM and n = 3.9 (approx. 4), suggesting a cooperative binding of 4 molecules of ligand. To summarize, planarians almost exclusively glided in normal conditions and, when exposed to AITC, gliding gaits were reduced in compensation for the nociceptive ‘scrunching’ gait induced by the chemical in a dose-dependent manner. Other undefined reactions were present (e.g., head turns, tail swings, C-shapes, etc.) but at low levels in various AITC conditions.

#### 3.1.2 Modulation by morphine and meloxicam

To try and modulate the nociceptive scrunching gait induced by AITC, we studied the effects of two common analgesics, morphine, and meloxicam. First, we applied them directly into the water together with various concentrations of AITC (acute exposure). Morphine did not induce any scrunching gait on its own (from 0.1 ± 0.1%, *n* = 19 to 0.3 ± 0.3%, *n* = 12, *p* = 0.74, Figure 3C). In 25 μM of AITC, 1 μM of morphine significantly diminished the amount of scrunching displayed by the worms (from 26.8 ± 8.4%, *n* = 11 to 4.0 ± 1.9%, *n* = 12; *p* < 0.005). However, at either 40, 50 or 100 μM of AITC, acute exposure to 1 μM of morphine did not show any significant reduction in the scrunching gait (at 40 μM: from 64.0 ± 4.5%, *n* = 12 to 65.2 ± 6.0%, *n* = 12, *p* = 0.88; at 50 μM: from 83.4 ± 4.2%, *n* = 11 to 73.8 ± 7.9%, *n* = 9, *p* = 0.4; at 100 μM: from 91.6 ± 4.0%, *n* = 10 to 82.8 ± 4.3, *n* = 11, *p* = 0.07). As for meloxicam, it did not induce any scrunching gait on its own either (from 0.1 ± 0.1%, *n* = 19 to 0.6 ± 0.6%, *n* = 9, *p* = 0.58, Figure 3D), but 1 μM of meloxicam also reduced the scrunching gaits induced by 25 μM of AITC (from 26.8 ± 8.4%, *n* = 11 to 9.7 ± 6.7%, *n* = 10, *p* < 0.05). At 50 μM of AITC however, meloxicam did not reduce the amount of scrunching gaits displayed (from 83.4 ± 4.2%, *n* = 11 to 88.6 ± 2.6%, *n* = 12, *p* = 0.56). Hence, when exposed acutely with small concentrations of AITC, morphine and meloxicam significantly reduced the amount of nociceptive behavior displayed by the worms. At higher concentrations of AITC, they did not manage to reduce these scrunching gaits.

Because morphine and meloxicam might not fully penetrate the outer layer of tissue of the animal within the 5 min of the test, we decided to take another approach. We therefore decided to bathe the animals for 2 h in 1 μM of either morphine or meloxicam. Baths of morphine did not induce any scrunching on its own (from 0.1 ± 0.1%, *n* = 19 to 0 ± 0%, *n* = 12, *p* = 0.47, Figure 3E), and it significantly reduced the nociceptive gait at both 25 μM (from 26.8 ± 8.4%, *n* = 11 to 0.9 ± 0.5%, *n* = 12, *p* < 0.001), and at 50 μM of AITC exposure (from 83.4 ± 4.2%, *n* = 11 to 59.9 ± 6.1%, *n* = 12, *p* < 0.005). The best fit by the same Hill model for morphine-exposed worms in AITC showed a shifted $EC_{50}$ from 34.65 μM to 43.64 μM (Figure 3B). At 100 μM, no difference could be seen anymore (from 91.6 ± 4.0%, *n* = 10 to 83.8 ± 3.9%, *n* = 12, *p* = 0.09). Baths of meloxicam showed similar results. Meloxicam did not induce any scrunching on its own either (from 0.1 ± 0.1%, *n* = 19 to 2.0 ± 2.0%, *n* = 12, *p* = 0.74, Figure 3F), and it also significantly reduced the nociceptive gait at both 25 μM (from 26.8 ± 8.4%, *n* = 11 to 9.6 ± 5.4%, *n* = 10, *p* < 0.05) and 50 μM of AITC exposure (from 83.4 ± 4.2%, *n* = 11 to 15.6 ± 7.4%, *n* = 10, *p* < 0.001). Thus, exposing worms for 2 h in morphine solutions improved its anti-nociceptive effect by reducing scrunching gaits induced by higher concentrations of AITC. Baths of meloxicam also allowed for a strong reduction of scrunching gaits in higher concentrations of AITC.

### 3.2 Heat avoidance

**Figure 4:**
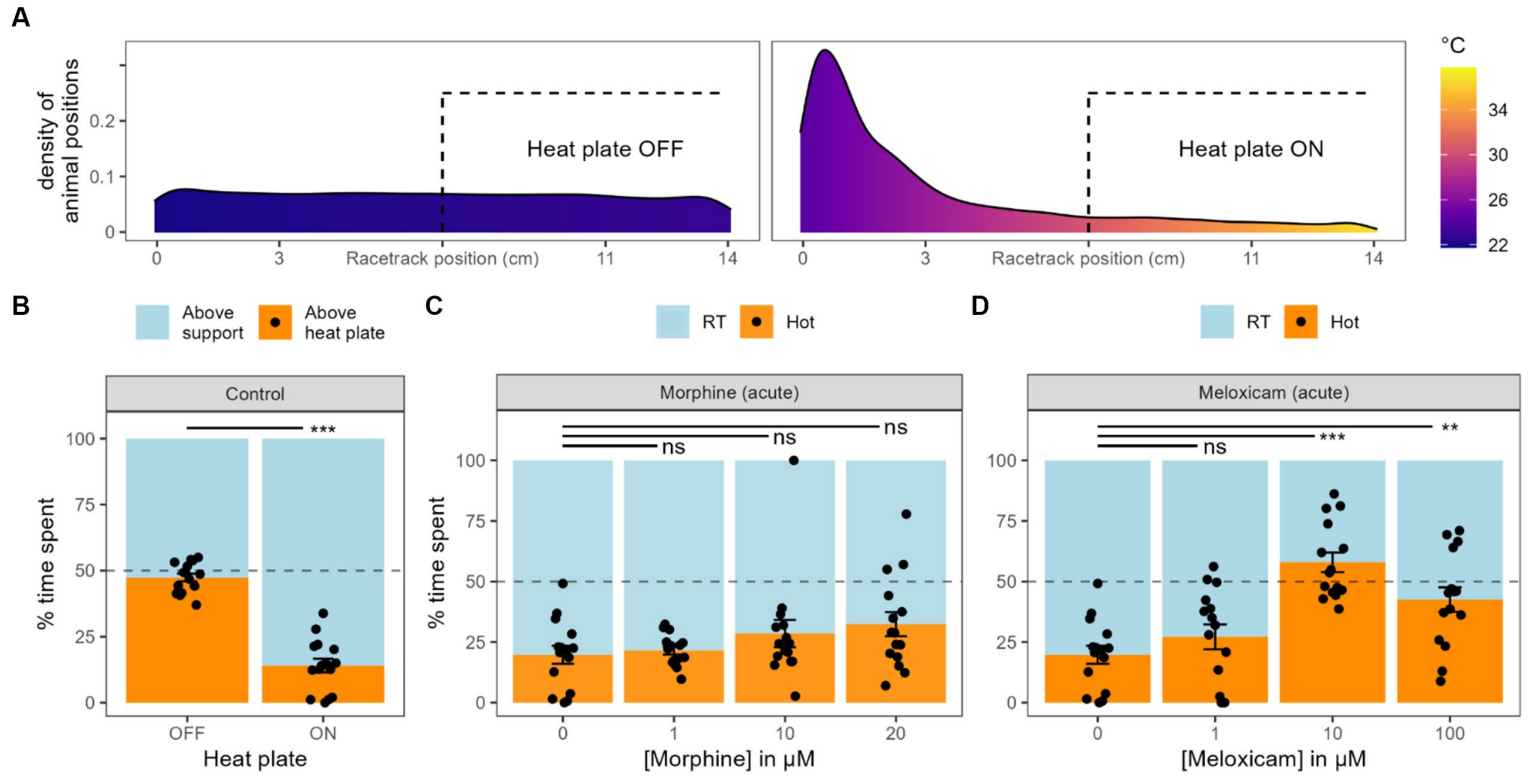
Heat avoidance. **(A)**. Density of animal position along the racetracks coordinates together with the associated temperatures, either when the heat plate is turned OFF or ON. **(B)** Graph bar equivalent of panel **A**, with the time spent by the worms on each side of the racetracks. Black dots represent individual animals, bars represent the mean and the error bars represent the SEM (also valid for next panels). **(C)** Effect of different concentrations of morphine acute exposure on heat avoidance. **(D)** Effect of different concentrations of meloxicam acute exposure on heat avoidance.

Planarians display negative thermotaxis, avoiding water temperature they are not acclimated to Arenas et al. (2017). Place preference in a range of temperature or heat avoidance are common tests used on rodents to study pain and nociception (Deuis et al., 2017). To adapt such heat avoidance test on planarians, we used racetracks (see Material & Methods), created a temperature gradient from 22°C to 36°C and tracked their position along the tracks. When the heat plate was off and the temperature was homogenous along the track, the worms spent equivalent time on each half, which did not differ from 50% randomness (47.4 ± 1.5%, *n* = 15, *p* = 0.14, Figures 4A,B). With the heat plate on, they spent significantly less time on the hotter half (14.0 ± 2.6%, *n* = 15, *p* < 0.001). In further analysis, the RT side and the hot side were not divided by the center of the racetracks. Instead, they were split by the midpoint of the min-max temperature range (see Materials and methods section). Using this correction, planarians spent on average 19.8 ± 3.7% (*n* = 15) on the hotter side.

#### 3.2.1 Modulation by morphine and meloxicam

Just as in chemical tests, we added morphine or meloxicam directly into the water during the place preference test (acute exposition). We tried various concentrations of morphine, but none of them made the worms spend significantly more time on the hot side compared to the control condition (1 μM: 21.5 ± 1.7%, *n* = 15, *p* = 0.69; 10 μM: 28.5 ± 5.6%, *n* = 15, *p* = 0.3; 20 μM: 32.4 ± 5.0%, *n* = 15, *p* = 0.07, Figure 4C). Analysis of variance did not display any significative trend either for the effect of morphine on the time spent in hot sides (*p* = 0.28). Meloxicam, on the other hand, did induce a change in heat avoidance at an exposure of 10 μM (57.9 ± 4.0%, *n* = 15, *p* < 0.001, Figure 4D) and 100 μM (42.5 ± 5.0%, *n* = 15, *p* < 0.01). At a concentration of 1 μM of meloxicam, the worms did not spend significantly more time in the hot side of the track (27.2 ± 5.1%, *n* = 15, *p* = 0.2).

We also exposed the worms to baths of various morphine or meloxicam concentrations for 2 h before the heat avoidance tests (Supplementary Figure S2). After 2 h in 1, 10 or 20 μM of morphine, they spent, respectively, 17.1 ± 5.0% (*n* = 15, *p* = 0.43), 1.5 ± 0.6% (*n* = 15, *p* < 0.001) and 19.8 ± 4.0% (*n* = 15, *p* = 0.77) of the test in the hot water, *p*-values representing the comparison with the water-only control. Thus, morphine did not induce any change in heat avoidance at 1 and 20 μM, but made planarians spend significantly less time in the hot water when pre-exposed to 10 μM, which was unexpected. For meloxicam, only a concentration of 1 μM could be tested, as higher concentrations for a prolonged exposure highly reduced the worms’ locomotion. At this concentration, they spent 23.1 ± 5.2% (*n* = 15, *p* = 0.59) in the hot side of the racetrack, which was not significantly different from the water-only control.

To summarize, acute exposure to morphine did not change the heat avoidance from worms in any condition, but meloxicam strongly reduced it at both 10 and 20 μM. When pre-exposed for 2 h in either morphine or meloxicam, planarians heat avoidance was not reduced in any condition we exposed them to.

### 3.3 Thigmotaxis

**Figure 5:**
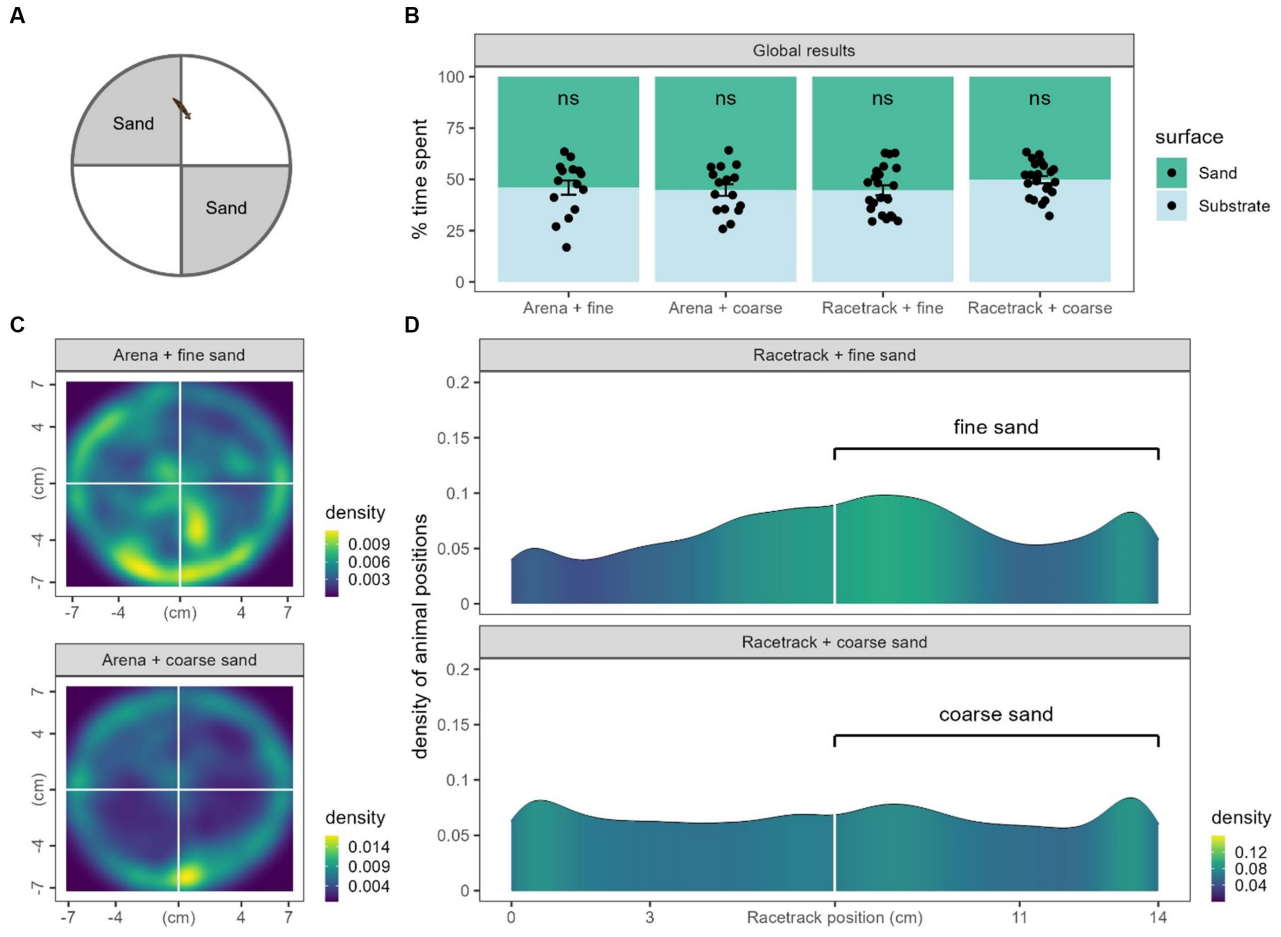
Thigmotaxis results. **(A)** Recall from figure to show what part of the arenas were covered in sand. **(B)** Global results of time spent on each surface for the four conditions from panel **C** and **D**. Black dots represent individual animals, bars represent the mean and the error bars represent the SEM. To observe significance, means were compared to randomness (50%). **(C)** 2D density of animal positions in arenas. Note that the lateral sides (walls) of the petri dishes were also covered in sand. **(D)** Density of animal positions along the racetracks coordinates.

In order to study mechanically-induced nociception in Gd, another place preference test was done using sand in either arenas or racetracks. Arenas were separated into 4 quarters and racetracks into two halves (see Materials and methods and Figure 1). We measured the time spent on each surface and, in any case, we did not observe any significant place preference for any substrate. No condition displayed a significantly different time spent in the smooth side than from 50% randomness (Arena + fine sand: 46.0 ± 3.4%, *n* = 15, *p* = 0.48; Arena + coarse sand: 44.8 ± 2.8%, *n* = 16, *p* = 0.09; Racetrack + fine sand: 44.65 ± 2.4%, *n* = 22, *p* = 0.06; Racetrack + coarse sand: 49.8 ± 1.8, *n* = 22, *p* = 0.9, Figure 5B). Because no preference or avoidance could be seen from the data, we also plotted the density of the animals positions to visually inspect them (Figures 5C,D). In circular arenas, it appears that there is a slightly higher preference for animals exposed to fine sand to explore the center of the dish than for animals exposed to coarse sand. However, they both still mostly glide against the edge of the dishes. Also note that the edges of the dishes were also covered in sand. In racetracks, we can also hardly interpret a slight (not significant) preference for the fine sand side, while the positions are homogenously distributed in the coarse sand condition.

Therefore, in our experimental conditions, it does not appear that Gd displayed any place preference or aversion regarding rough surfaces.

### 3.4 RNAi: TRPA1 knockdown

TRPA1 ion channels are crucial molecular players in both chemical and thermal nociception and are well conserved throughout the whole animal kingdom (Laursen et al., 2015). Notably, one strong naturally-occurring agonist of TRPA1 ion channels is AITC (Bandell et al., 2004). With this in mind, we knocked-down the expression of TRPA1 using RNAi technology and tested the worms in both chemical and thermal nociceptive tests.

In chemical test settings, we exposed Gd-TRPA1 RNAi worms to a concentration of 50 μM of AITC. Compared to untreated worms, inhibition of TRPA1 expression induced a drastic change in displayed reactions with gliding gaits reaching levels comparable to non-AITC controls (from 11.6 ± 3.8%, *n* = 11 to 96.0 ± 1.9%, *n* = 17, *p* < 0.001, Figure 2C) and scrunching gaits dropping to zero (from 83.4 ± 4.2%, *n* = 11 to 0 ± 0%, *n* = 17, *p* < 0.001). A group of GFP RNAi worms was also used as a control. Compared to untreated worms, the GFP RNAi worms did not show any change in the amount of gliding (11.6 ± 3.8%, *n* = 11 vs. 19.0 ± 4.8%, *n* = 14; *p* = 0.18), nor in scrunching (83.4 ± 4.2%, *n* = 11 vs. 79.9 ± 5.1%, *n* = 14; *p* = 0.83).

In heat avoidance tests however, Gd-TRPA1 RNAi animals did not display any change in place preference to the heat (20.0 ± 3.5%, *n* = 20, Figure 2D) compared to either untreated worms (19.8 ± 0.7%, *n* = 15, *p* = 1) or GFP RNAi worms (25.8 ± 2.5%, *n* = 15, *p* = 0.2).

In summary, Gd-TRPA1 knockdowns annihilated the nociceptive scrunching behaviors, making planarians glide normally even in high AITC concentrations. However, and surprisingly, Gd-TRPA1 knockdowns did not induce any change in heat avoidance in our experimental setup.

## 4 Discussion

In this study, we exposed planarians to three different types of nociceptive stimuli: chemical, thermal and mechanical. We also focused on modulating responses to these stimuli using common pain-killers in this multimodal paradigm.

### 4.1 Chemical nociception

First, to observe the effect of chemically-induced nociceptive behaviors, we adapted the methodology used by Sabry et al. observing the scrunching gait they extensively described in 2015 (Cochet-Escartin et al., 2015; Sabry et al., 2019). We observed planarian behavior for a prolonged period of time (5 min) because chemically-induced scrunching showed to induce high amounts of undefined reactions in the first minute of exposure and robust gaits later on (see Supplementary Figure S1). Doing so, we managed to show that Gd displayed a strongly reproducible scrunching gait in the presence of AITC. We could also demonstrate that this chemically-induced nociceptive gait is dose dependent and that it could be modeled by a classical Hill dose–response curve. It should be emphasized that the induction of the scrunching gait is in compensation for a diminished normal gliding gait: other undefined noxious gaits were observed but in a constant manner and in very low amount.

In order to further explore the mechanisms of this chemically-induced nociceptive behavior, we focused our analysis on TRPA1 ion channels. This receptor is of particular interest because it is implicated in both chemical nociception – with AITC being a potent agonist - and in thermal nociception. It is known, through *in situ* hybridization, that at least some planarian species express TRPA1 ion channels in sensory neurons, mostly located around the head of the animal in Dj (Inoue et al., 2014). Using RNAi technology based on the insertion of dsRNA into the worms’ food, we managed to knockdown Gd-TRPA1 expression by more than 70% (Figure 2B). When we exposed Gd-TRPA1 RNAi planarians to 50 μM of AITC, they were gliding smoothly, just as in freshwater, and no scrunching could be seen at all (Figure 2C). The fact that the knockdown was sufficient to fully suppress the scrunching gait revealed the implication of the TRPA1 receptor activation in the scrunching response to AITC at this concentration. This causal relationship was already shown in other planarian species such as *Schmidtea mediterranea* (Smed) and *Dugesia japonica* (Dj) (Sabry et al., 2019). By independently demonstrating the same causal link in Gd, we showed that the mechanisms of scrunching (i.e., nociceptive behavior) are well conserved across planarian species.

### 4.2 Heat avoidance

Apart from chemicals, another common use of nociceptive stimuli is through heat, either as a noxious reaction inducer (e.g., tail flick or hot plate test for rodents), or as an unpleasant condition to avoid in a place preference test (Deuis et al., 2017). We know that planarians display negative thermotaxis: they strongly avoid temperatures above 25–30°C, they thrive around 15–25°C, and they crimple below 15°C (Inoue et al., 2014). In this study, we limited the temperature range from RT (~22–24°C) to hot (~34–36°C) and showed that Gd also display net avoidance reactions to hot water, spending most of their time on the RT side.

As previously mentioned, TRPA1 ion channels are also highly involved in thermal nociception. However, interestingly, our study did not show evidence of the lack of thermotaxis when TRPA1 receptors were knocked-down. In a study by Arenas et al., although they used the same temperature ranges (22–34°C), they showed clear disruption of heat avoidance in Smed-TRPA1 knockdowns (Arenas et al., 2017). They used the species Smed and we chose Gd, but results are strikingly different as we did not show any change in heat avoidance at all. Similarly, in another recent study by Sabry et al., knocking down Dj-TRPA1 did not reduce the heat-induced scrunching gaits in Dj (Sabry et al., 2019). It can be noted that, although TRPA1 seems to be the main effector of the thermal response in these species, planarians also possess TRPV1 receptors, which are known for being thermosensitive as well (Sabry et al., 2019). Another idea developed by this team is the involvement of reactive oxygen species (ROS) as an intermediate created by heat and in turn activating TRPA1 ion channels (Arenas et al., 2017). This is corroborated by the fact that UV-light, known to induce ROS production, can also induce body contractions in planarians, and that inhibition of TRPA1 expression also inhibits these UV-induced reactions (Birkholz and Beane, 2017). However, that does not explain the difference between species, especially since UV light induces scrunching in Dj and not in Smed (Sabry et al., 2020). Also, while TRPA1 ion channels are highly conserved across the animal kingdom, its range of activation broadly varies between species (Laursen et al., 2015). The fact that Gd-TRPA1 RNAi planarians can still display scrunching gaits or heat avoidance strongly suggests that other receptors may be involved in the heat avoidance response.

### 4.3 Modulation by morphine and meloxicam

In order to justify the use of planarians as a nociception model, we should be able to modulate their reactions to judge the efficacy of a drug of interest. Here, we focused on commonly used antinociceptives, such as the gold standard pain killer morphine (Paul et al., 2021), which had already been used multiple times over the last few decades to modulate planarian behavior (Reho et al., 2022). In our study, the observed lack of antinociceptive properties of morphine when co-administered with high concentrations of AITC was not surprising. Indeed, morphine acts on various opioid receptors, which are G-protein coupled receptors (GPCRs), thus the time of action of morphine in planarians could be slower than the 5 min of exposition that we used (Gades et al., 2000). When worms were bathed in morphine for 2 h prior to experimental testing however, morphine showed high antinociceptive effects on the worms behavior, even at the higher AITC concentrations of 50 μM. At 100 μM, there was no reduction in scrunching anymore, but AITC concentration reached a high plateau of induced reactions, suggesting a saturation and thus a reduced potential effect for morphine. Even though opioid receptors have not yet been described in planarians to the best of our knowledge, a strong suggestion that some form of opioid receptor may be present is the presence of an endogenous opioid, met-enkephalin, in planarian neurons (Venturini et al., 1983).

To explore other potential antinociceptive impacts of morphine on planarians such as on thermal nociception, we also exposed them to morphine in the thermal place preference assays. Morphine’s impact on thermal sensitivity is known, especially in rodents, but to the best of our knowledge has never been tested on planarians (Morin et al., 1999; Morgan et al., 2012). Despite seemingly trending towards a significant effect, our results did not show any significative modulation by morphine on thermal place preference.

Besides morphine, overall similar effects were seen when exposing planarians to meloxicam, an NSAID. Indeed, our study showed that acutely exposing worms to meloxicam did not reduce the amount of scrunching observed but bathing the worms in meloxicam for 2 h before the test showed a great antinociceptive action, highly reducing the noxious scrunching gaits displayed by the worms. The first and only yet study that explored the effects of meloxicam on planarian nociception was done by Kim and Rawls in which they observed that meloxicam was able to reduce noxious “C-shapes” reactions induced by acute nicotine exposure (Kim and Rawls, 2022).

NSAIDs are mainly used to reduce inflammation, but are known to also modulate thermal sensitivity (Dogrul et al., 2007). We could not find any study that exposed planarians to another NSAID in a thermotaxis essay. Henry et al. set up a protocol highlighting planarian thermotaxis and showed that ibuprofen, another NSAID, managed to modulate thermotaxis on zebrafish, but unfortunately did not expose planarians to it (Henry et al., 2022). In our study, not only did we show that meloxicam could reduce noxious reactions induced by an irritant, but that it also managed to modulate planarian heat avoidance, making them prone to spend more time in hotter conditions.

### 4.4 Thigmotaxis

Finally, we also explored mechanical nociception in Gd. Planarians are assumed to be immune to cuts of parts of their body because of their remarkable regenerating capabilities (Reddien, 2018), though it does not prevent them from properly sensing their environment, from adapting to various surfaces or from simply avoiding threatening situations. Indeed, together with chemotaxis and thermotaxis, thigmotaxis and rheotaxis have been observed in planarians long ago (Pearl, 1903). Moreover, they display strong aversive reactions when abruptly cut in half and generally display aversive reactions (contractions, elongations, avoidance) when poked or touched, even gently (Cochet-Escartin et al., 2015; Le et al., 2021, see also Supplementary Video S1). However, cutting parts of Gd is not ideal to quantify behaviors as they react for a limited time of only a few seconds. This in mind, we adapted an experiment from Inoue et al. and Mohammed Jawad et al. where we exposed planarians to sharp surfaces and quantified their place preference (Inoue et al., 2015; Mohammed Jawad et al., 2018). In the study by Inoue et al. (2015) Dj had a tendency to stay on smooth surfaces and avoided rough scratched plastic. We assessed the hypothesis that scratched plastic could be, beyond thigmotaxis, actually aversive due to its roughness and the presence of small sharp and prickly pieces of plastic that could be detected by nociceptive mechanoreceptors. Preliminary tests we did with Gd showed that they did not avoid scratched plastic sides at all, so we adapted another place preference protocol from Mohammed Jawad et al. using sand to texture the substrate with sharp surfaces (Mohammed Jawad et al., 2018). In another pre-print study that used sand to texture sides of petri dishes, Dj preferred the sand-textured sides over the smooth petri dish side (Samuel et al., 2021). In our study, using Gd, no preference could be determined for any condition, even though we tend to see a small preference for sand textured surfaces. One hypothesis for this lack of avoidance is that Gd produce high amounts of mucus protecting them from predators and dryness, which also makes them stickier than Dj and Smed (Ireland et al., 2020; Goel et al., 2022), and given the bigger size of Gd, it might also better protect them from close-by sharp surfaces. Because these results differ from those of the literature, we assumed that it would be relevant to discuss them. However, the study of mechanical nociception in planarians could still benefit from new ideas and protocols to be properly quantified.

## 5 Conclusion

This study highlighted new anti-nociceptive effects of morphine and meloxicam in a multimodal fashion, testing multiple paradigms of nociception in novel and adapted nociceptive tests for planarians. Chemically-induced nociceptive gaits also showed great reproducibility in concordance with the literature and we confirmed the involvement of TRPA1 receptors on the scrunching response induced by AITC in *G. dorotocephala*, which was already seen in other species. Thermal and mechanical tests, however, showed discrepancy with the literature and would be of interest to be further explored.

## Data availability statement

The original contributions presented in the study are included in the article/Supplementary material, further inquiries can be directed to the corresponding author/s.

## Author contributions

GR: Validation, Supervision, Software, Writing – review & editing, Writing – original draft, Visualization, Project administration, Methodology, Investigation, Funding acquisition, Formal analysis, Data curation, Conceptualization. YM: Formal analysis, Investigation, Data curation, Writing – review & editing, Resources, Methodology, Conceptualization. YG: Writing – review & editing, Methodology, Data curation, Conceptualization. VL: Supervision, Methodology, Writing – review & editing, Conceptualization. HC: Formal analysis, Writing – review & editing, Writing – original draft, Validation, Supervision, Resources, Project administration, Methodology, Funding acquisition, Conceptualization.
