# Supplementary figures and images for: Behavioral and pharmacological characterization of planarian nociception
Source: Front Mol Neurosci. 2024 May 1;17:1368009. doi: 10.3389/fnmol.2024.1368009 (PMC11094297; doi:10.3389/fnmol.2024.1368009)

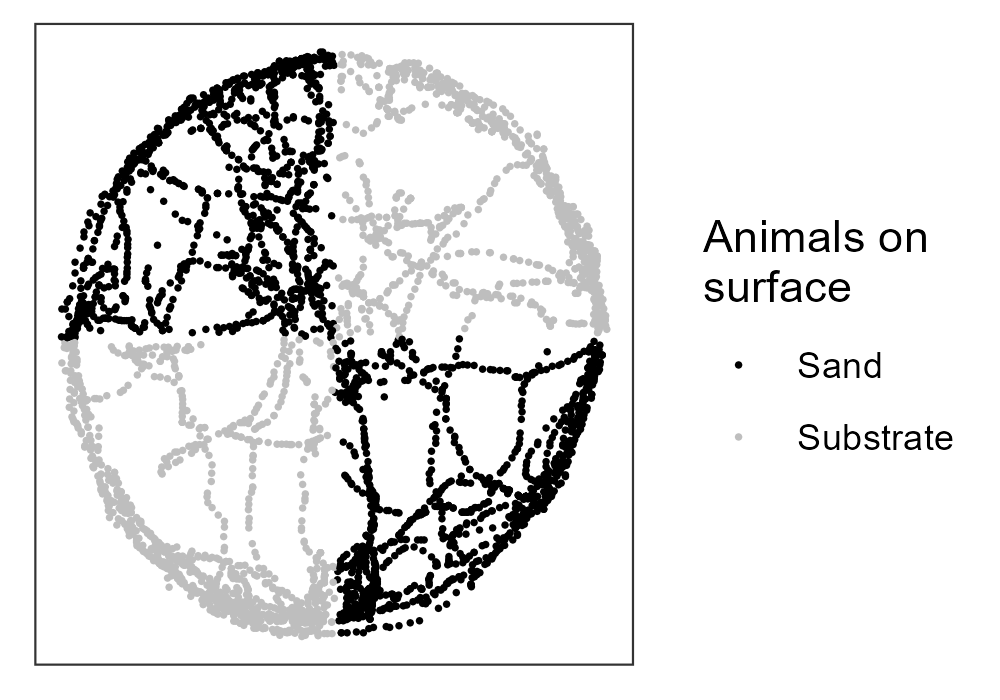

Supplement: Supplementary file 1 [file Data_Sheet_1.ZIP › Article planarian nociception - code and data/figures/fig 1.png]

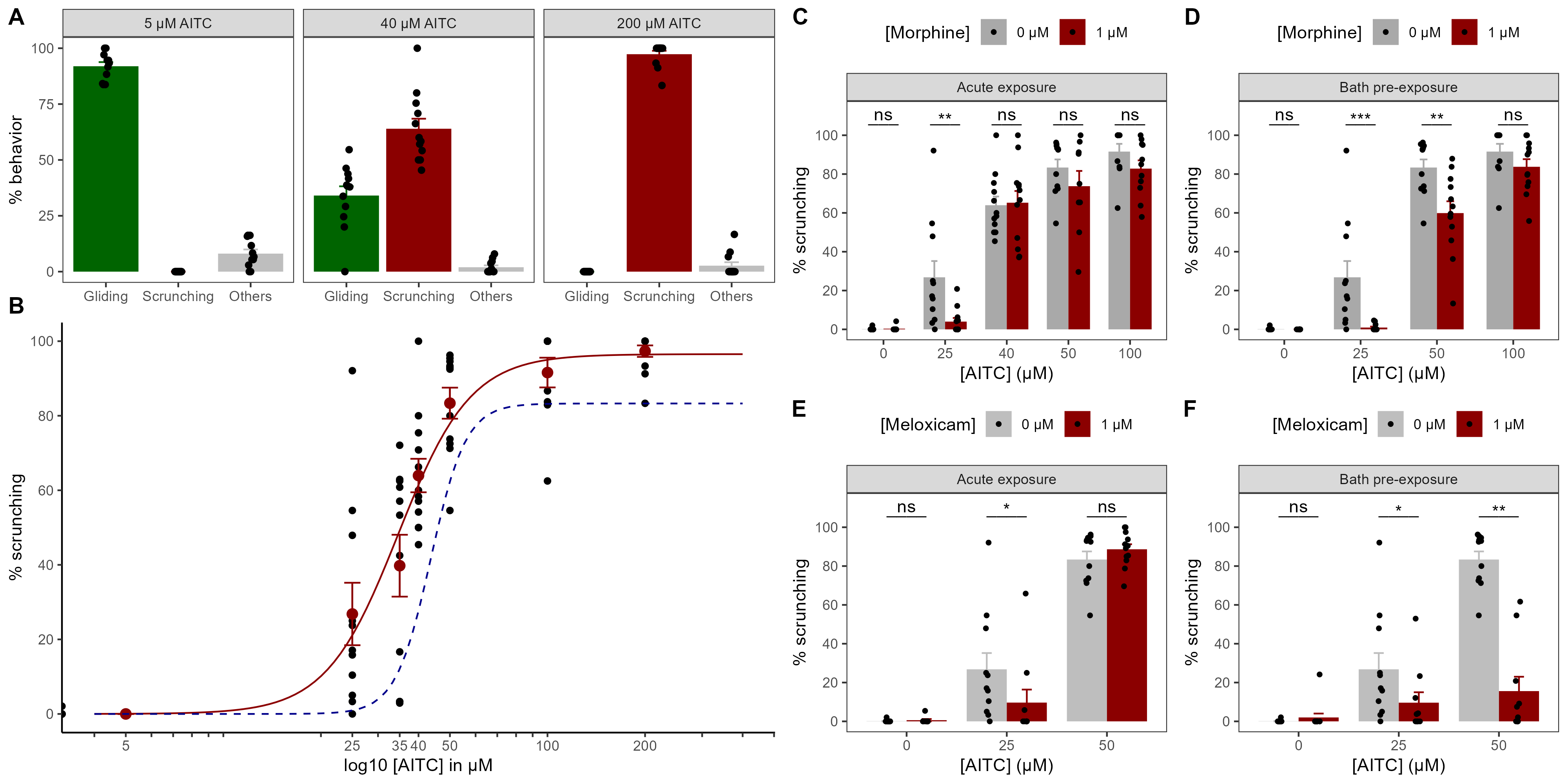

Supplement: Supplementary file 1 [file Data_Sheet_1.ZIP › Article planarian nociception - code and data/figures/fig 2.png]

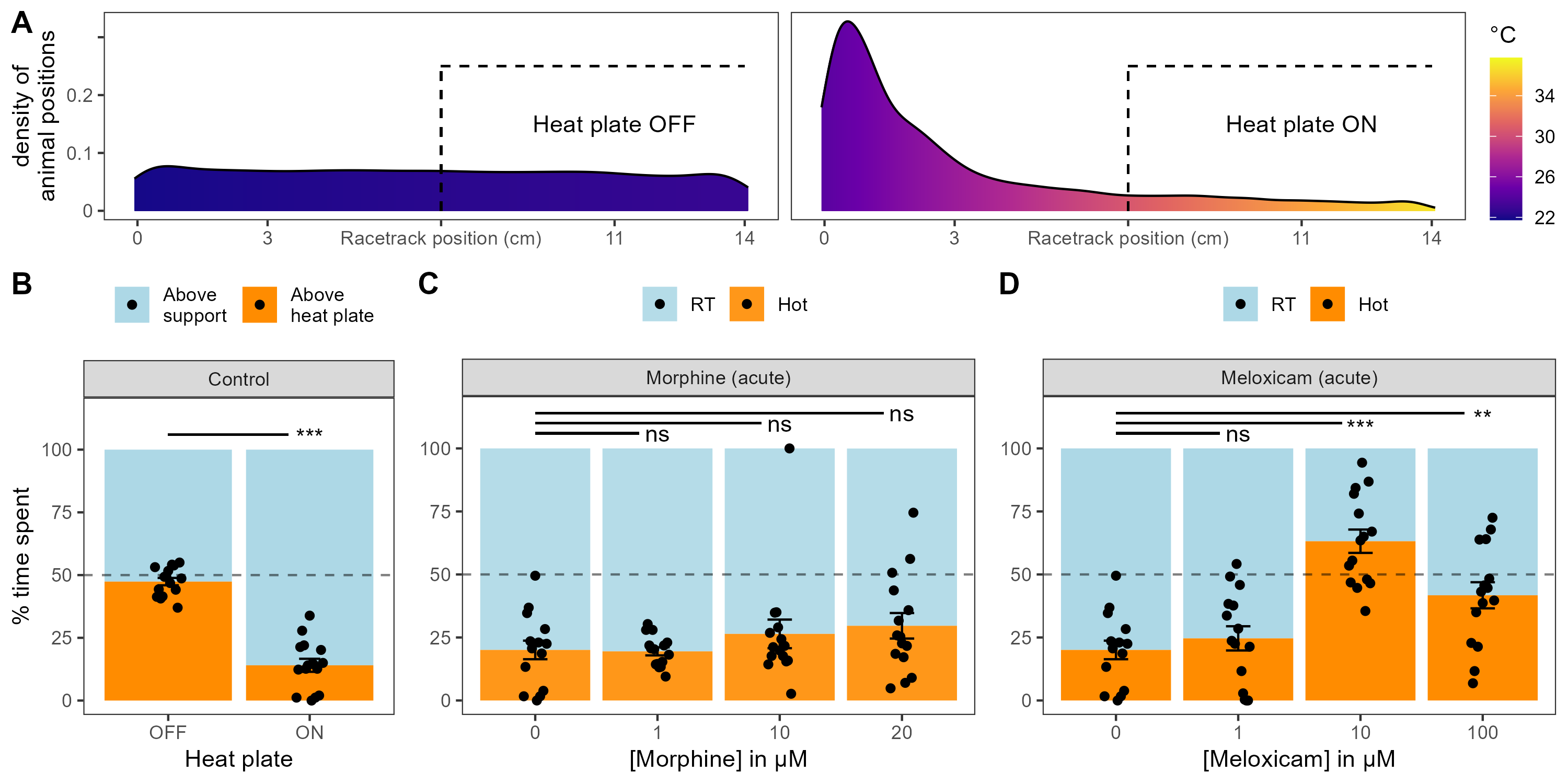

Supplement: Supplementary file 1 [file Data_Sheet_1.ZIP › Article planarian nociception - code and data/figures/fig 3.png]

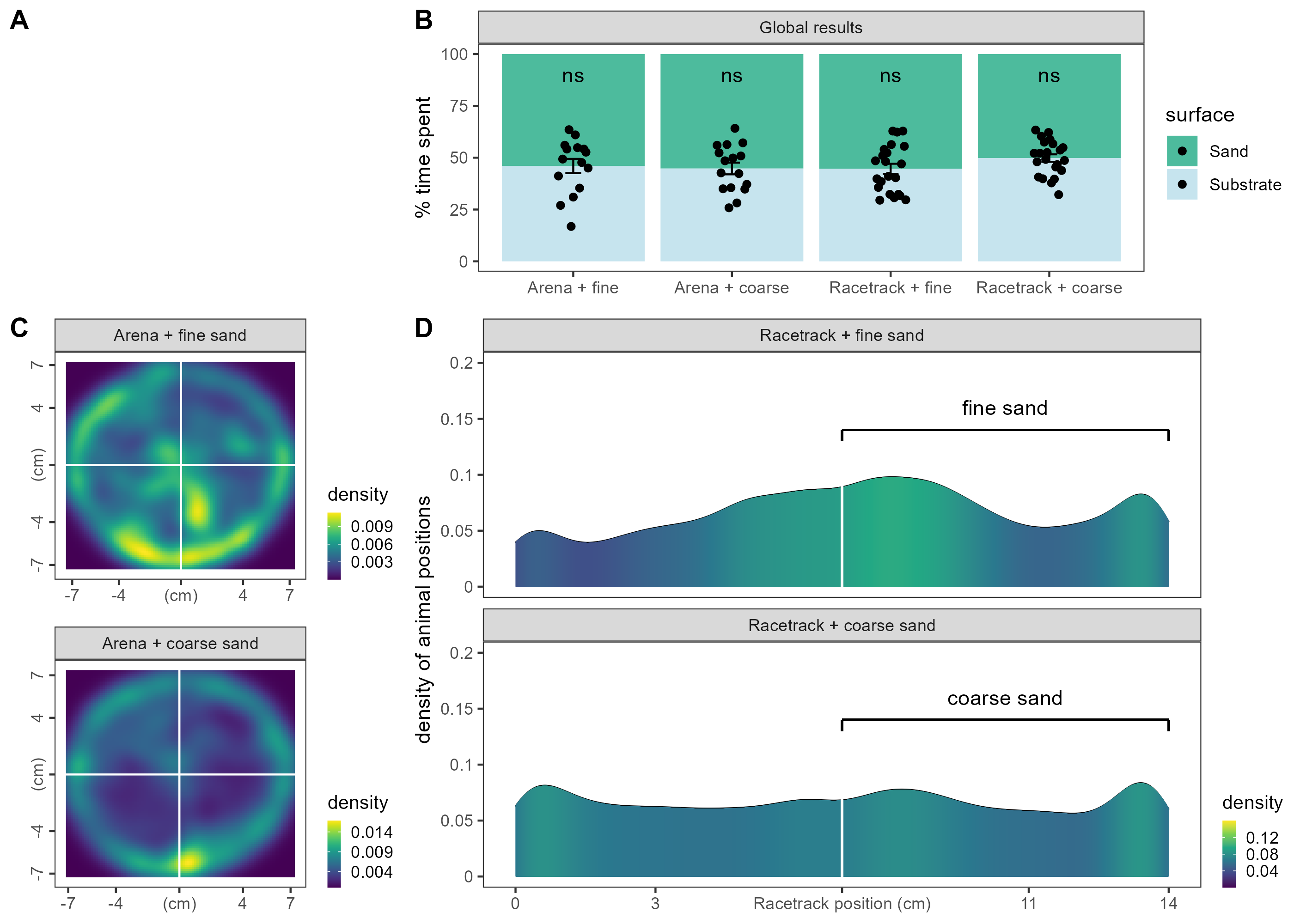

Supplement: Supplementary file 1 [file Data_Sheet_1.ZIP › Article planarian nociception - code and data/figures/fig 4.png]

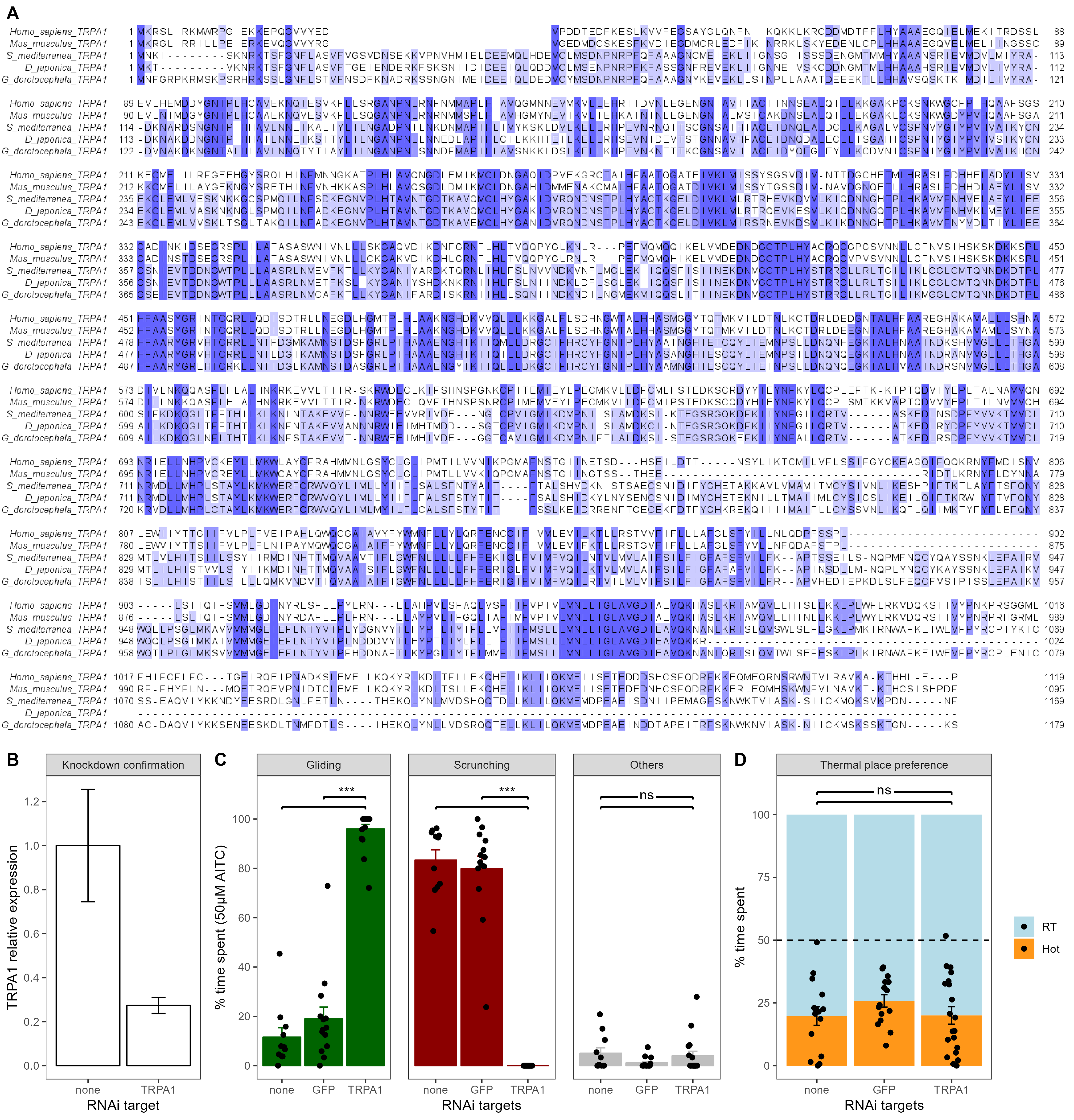

Supplement: Supplementary file 1 [file Data_Sheet_1.ZIP › Article planarian nociception - code and data/figures/fig 5.png]

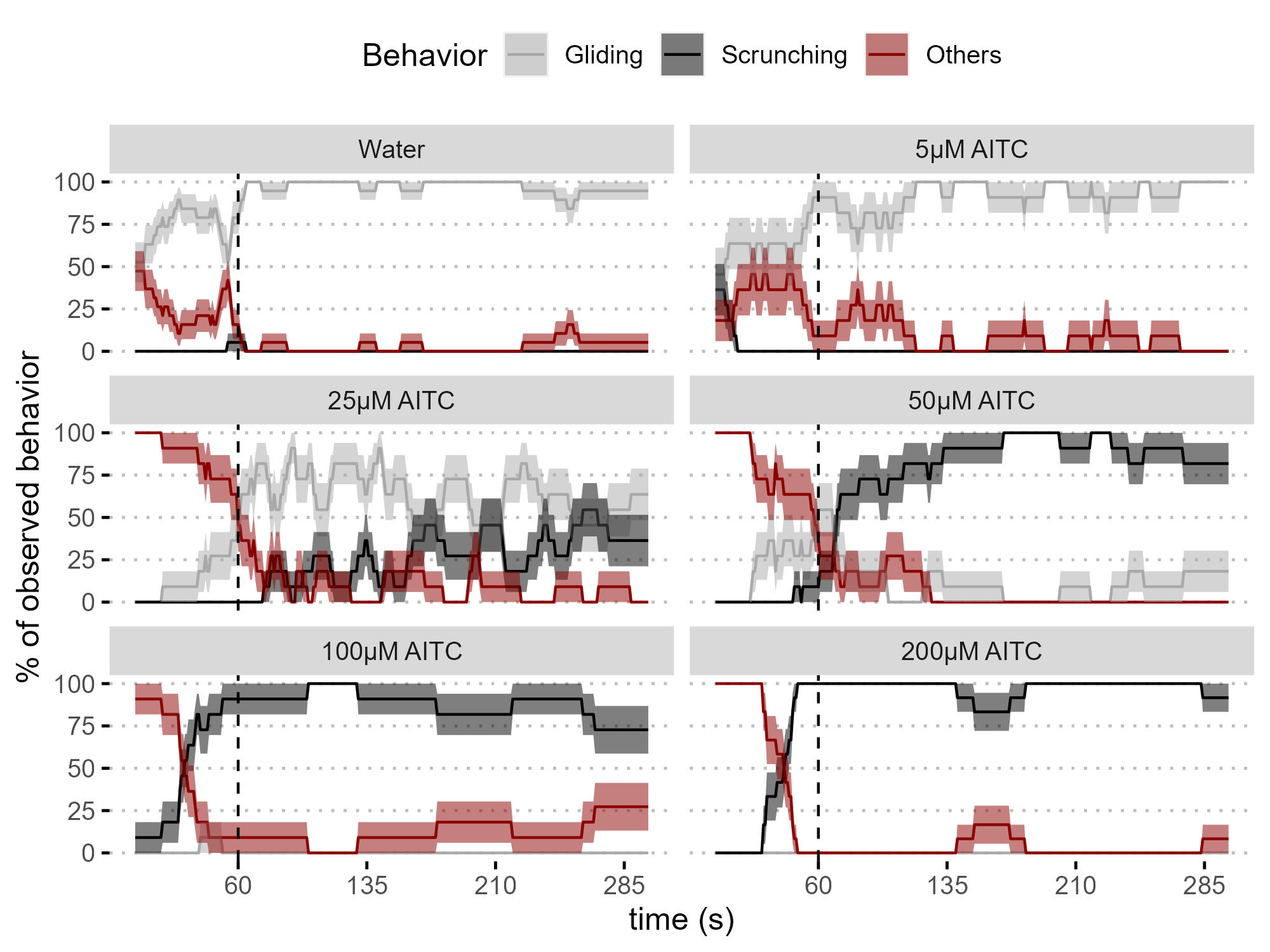

Supplement: Supplementary file 1 [file Data_Sheet_1.ZIP › Article planarian nociception - code and data/figures/fig supp 1.jpeg]

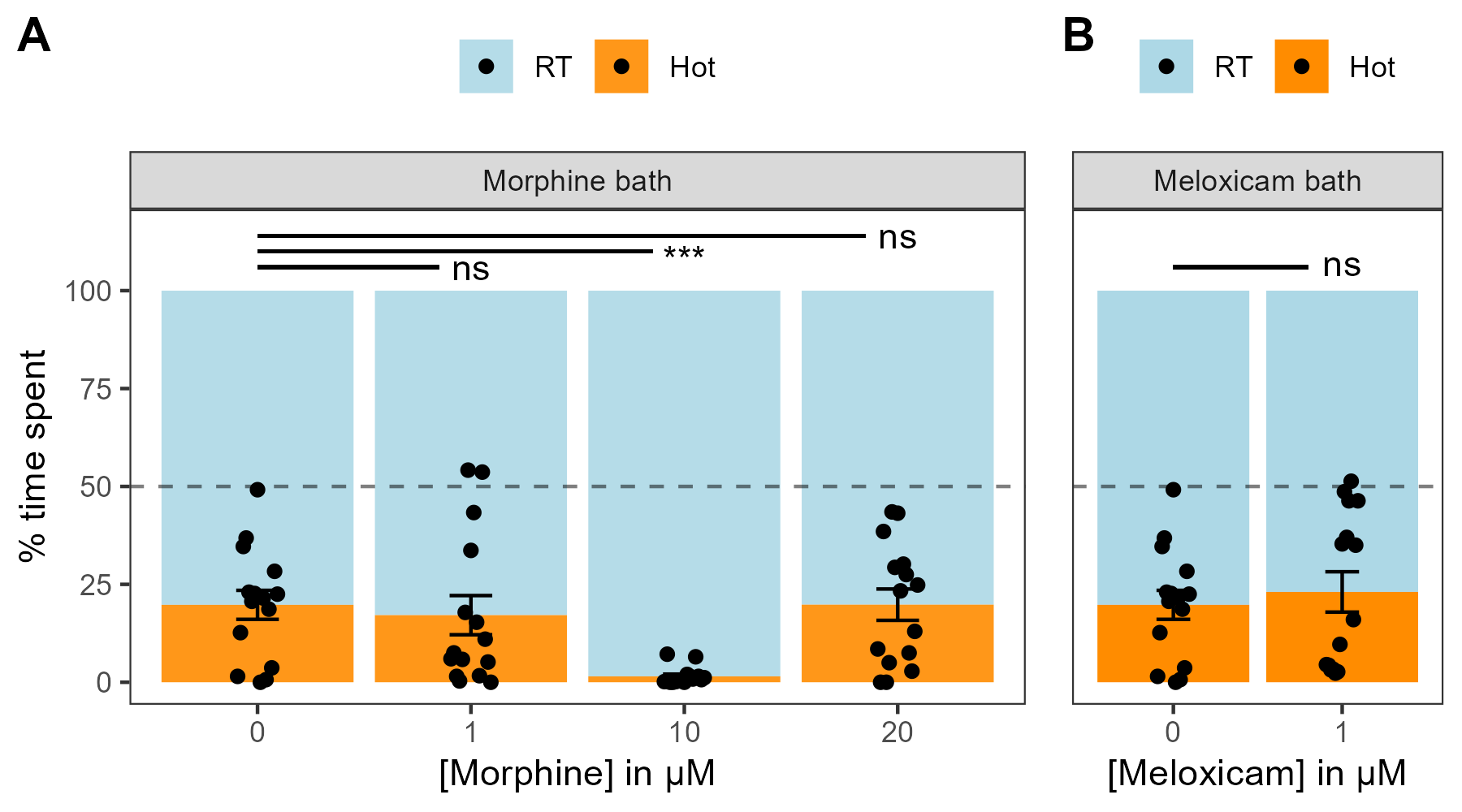

Supplement: Supplementary file 1 [file Data_Sheet_1.ZIP › Article planarian nociception - code and data/figures/fig supp 2.png]

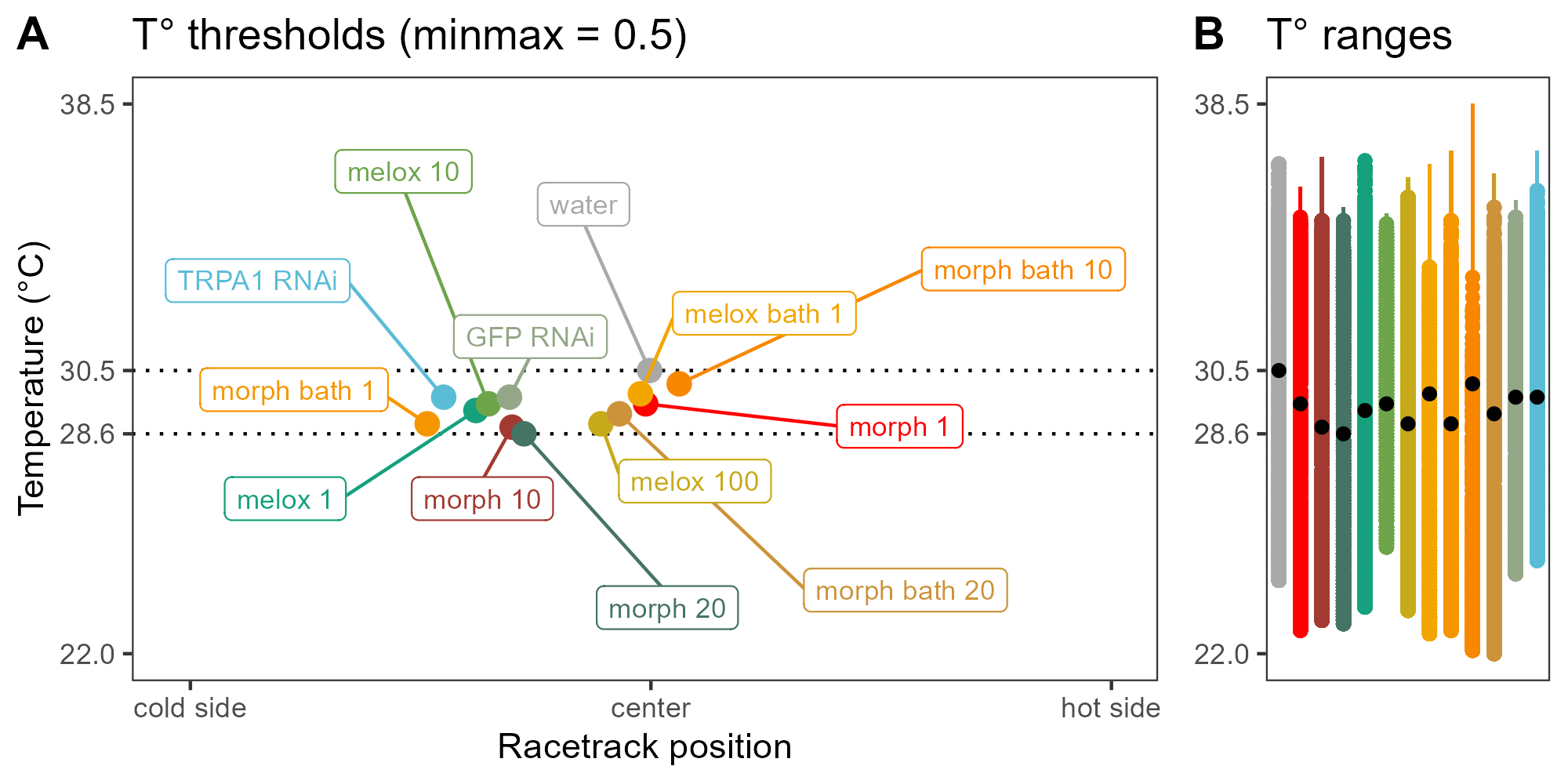

Supplement: Supplementary file 1 [file Data_Sheet_1.ZIP › Article planarian nociception - code and data/figures/fig supp 3.png]

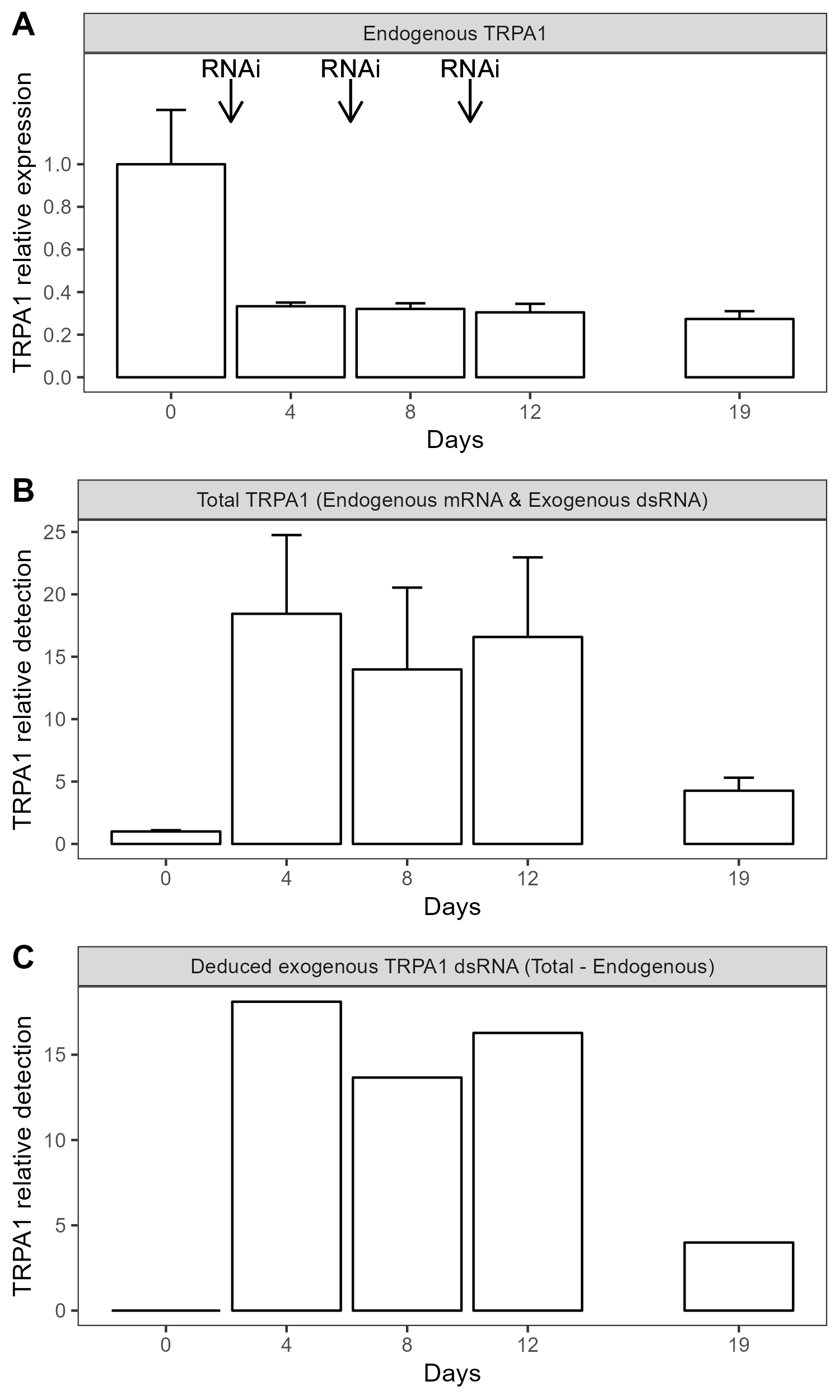

Supplement: Supplementary file 1 [file Data_Sheet_1.ZIP › Article planarian nociception - code and data/figures/fig supp 4.png]

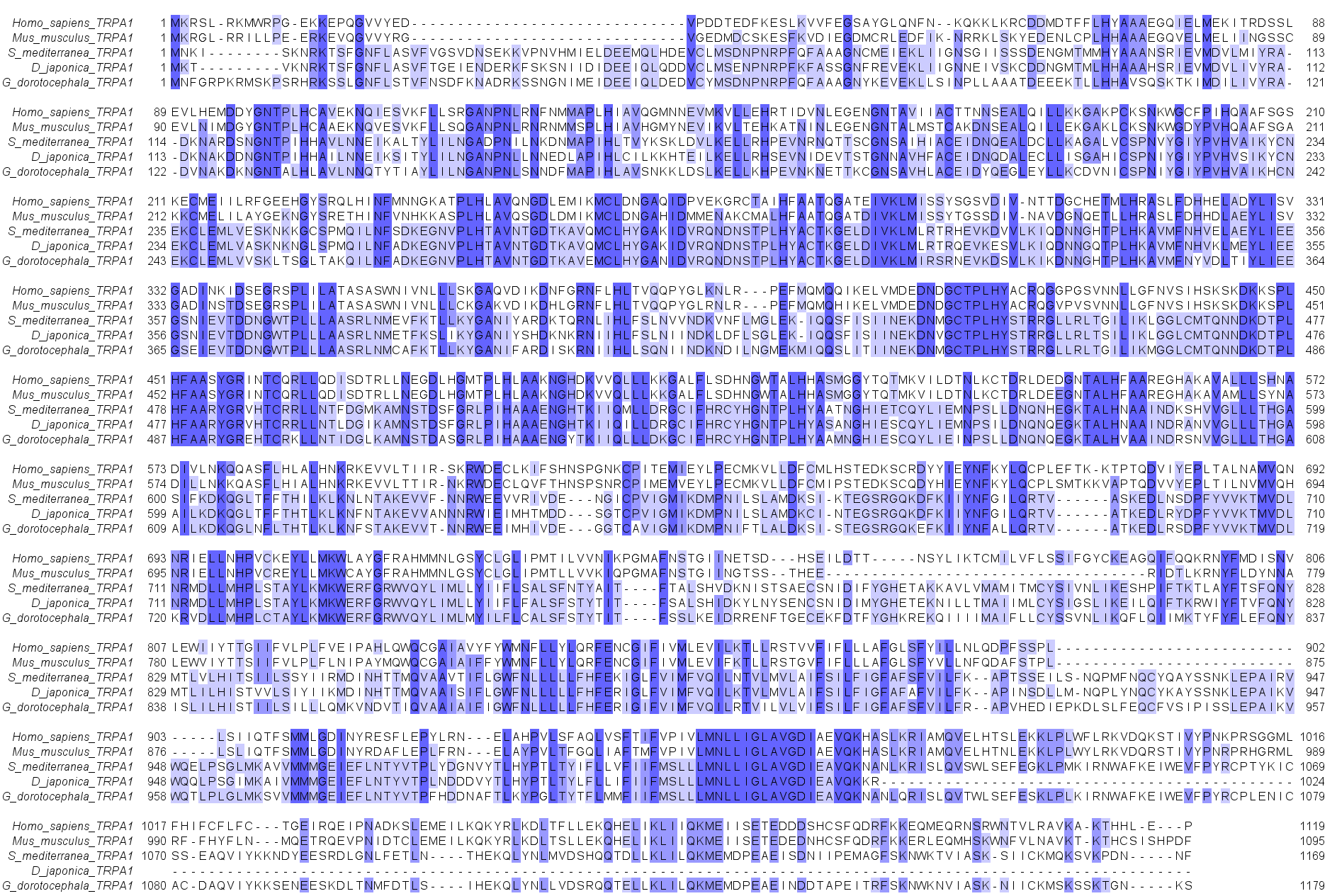

Supplement: Supplementary file 1 [file Data_Sheet_1.ZIP › Article planarian nociception - code and data/figures/sequence.png]
